# Herpes simplex virus 1 targets IRF7 via ICP0 to limit type I IFN induction

**DOI:** 10.1038/s41598-020-77725-4

**Published:** 2020-12-17

**Authors:** David Shahnazaryan, Rana Khalil, Claire Wynne, Caroline A. Jefferies, Joan Ní Gabhann-Dromgoole, Conor C. Murphy

**Affiliations:** 1 Department of Ophthalmology, Royal College of Surgeons in Ireland, Dublin 2, Ireland; 2 Department of Ophthalmology, Royal Victoria Eye and Ear Hospital, Dublin 2, Ireland; 3 School of Pharmacy and Biomolecular Sciences (PBS) and RSCI Research Institute, Royal College of Surgeons in Ireland, Dublin 2, Ireland; 4 School of Biological and Health Sciences, Technological University (TU) Dublin, Kevin Street, Dublin 8, Ireland; 5 Department of Medicine, Division of Rheumatology and Department of Biomedical Sciences, Cedars-Sinai Medical Centre, 8700 Beverly Blvd, Los Angeles, CA 90048 USA

**Keywords:** Immune evasion, Eye diseases, Corneal diseases, Pattern recognition receptors, Immunology

## Abstract

Herpes simplex keratitis (HSK), caused by herpes simplex virus type 1 (HSV-1) infection, is the commonest cause of infectious blindness in the developed world. Following infection the virus is initially suspended in the tear film, where it encounters a multi-pronged immune response comprising enzymes, complement, immunoglobulins and crucially, a range of anti-viral and pro-inflammatory cytokines. However, given that HSV-1 can overcome innate immune responses to establish lifelong latency throughout a susceptible individual’s lifetime, there is significant interest in understanding the mechanisms employed by HSV-1 to downregulate the anti-viral type I interferon (IFN) mediated immune responses. This study aimed to investigate the interactions between infected cell protein (ICP)0 and key elements of the IFN pathway to identify possible novel targets that contribute to viral immune evasion. Reporter gene assays demonstrated the ability of ICP0 to inhibit type I IFN activity downstream of pathogen recognition receptors (PRRs) which are known to be involved in host antiviral defences. Further experiments identified interferon regulatory factor (IRF)7, a driver of type I IFN, as a potential target for ICP0. These findings increase our understanding of the pathogenesis of HSK and suggest IRF7 as a potential therapeutic target.

## Introduction

Herpes simplex keratitis (HSK) is the commonest cause of infectious corneal blindness in the western world^1^. There are over 500,000 affected individuals in the United States (US) alone^2^. The infection is caused by human herpes simplex virus (HSV)-1 in the majority of cases, with the exception of neonatal herpetic keratitis, where 75% of cases is caused by HSV-2^3^. It is characterised by recurrent episodes of infection and inflammation in the cornea, which can result in significant corneal scarring and vision loss^4,5^. Although a significant amount of research has resulted in a better understanding of the molecular biology and pathogenesis of HSV-1, the herpetic eye infection remains a serious public health problem due to its significant impact on vision-related and general health-related quality of life.

Production of type I interferon (IFN-I) downstream of viral detection is a key component of the innate immune antiviral response^6^. In addition to Toll-like receptor (TLRs), retinol-inducible gene-I (RIG-I)-like receptor (RLR) and nucleotide-binding oligomerization domain (NOD)-like receptor (NLRs), cytosolic DNA sensors are also involved in the recognition of HSV-1. During HSV-1 infection, pattern recognition receptors (PRRs) including TLRs, RLRs and NLRs, recognise structurally conserved pathogen-associated molecular patterns (PAMPs) and trigger the production of type I interferon (IFN-I) and other pro-inflammatory cytokines^6^. TLR-mediated signalling through the downstream adapters Toll/IL-1R domain-containing adaptor-inducing IFN-β (TRIF) and myeloid differentiation factor 88 (MyD88) leads to activation of interferon regulatory factors (IRFs) and nuclear factor-kappa B (NFκB). RIG-I and melanoma differentiation-associated protein 5 (MDA5) detect distinct viral RNA structures and signal through the adaptor protein mitochondrial antiviral signaling protein (MAVS, or IPS-1/VISA/Cardif) resulting in IRF3 and NF-κB activation. RIG-I is an intracellular receptor for double-stranded RNA (dsRNA) viruses and as HSV-1 is a DNA virus, would not be expected to recognise double-stranded DNA (dsDNA) of HSV-1. However, it is known that HSV-1 synthesises dsRNA as a by-product of viral replication enabling its detection by RIG-1^7^. Additionally HSV-1 replicates more robustly in human hepatoma cells line lacking a functional RIG-I, suggesting a link between HSV-1 and RIG-I^8,9^. More recently several cytosolic DNA sensors have also been identified, including DNA-dependent activator of IFN regulatory factors (DAI), interferon gamma inducible protein 16 (IFI16), RNA polymerase III (Pol III), DEAD box helicase 41 (DDX41), and cyclic GMP-AMP synthase (cGAS), that contribute to initiation of host immune response upon detection of viral nucleic acids^10^. cGAS, IFI16 and DDX41 signal through a common adaptor molecule known as Stimulator of IFN genes (STING). STING functions to recruit and activate TANK-binding kinase 1 (TBK) culminating in the activation of IRF3 and the induction of type I IFNs.

During evolution many viruses have developed mechanisms to evade host responses and specifically the production of IFN-I by targeting different components downstream of the PRRs and cytosolic nucleic acid receptors^11^. IFN-I is essential to limit HSV-1 replication in the cornea as well as being required to limit the systemic spread of infection^12^, and HSV-1 has evolved multiple strategies to evade the host immune response in order to establish latency^13,14^. The HSV-1 encoded protein, infected cell protein 0 (ICP0), has been studied extensively in this regard, as it has been implicated in the pathogenesis of HSV-1 infection. ICP0 is a nuclear phosphoprotein that plays a crucial role in multiple aspects of the viral life cycle, including transactivation of HSV-1 gene expression^15^, initiation of lytic infection^16–18^ and establishment of^19^, and reactivation from, a latent viral state^20–22^. Infection of cultured cells with an immediate early (IE) gene-deficient HSV-1 mutant which does not encode ICP0 is known to lead to complete repression of the viral genome and establishment of a quiescent state, with the only method of reactivation being to reintroduce ICP0 by superinfection^23–26^.

ICP0, an E3 ubiquitin ligase, has developed various mechanisms to avoid immune-surveillance and promote viral replication. Studies have revealed that ICP0 induces proteasome-dependent degradation of the promyelocytic leukemia protein (PML) and Sp100 (speckled, 100 kDa) components of nuclear bodies^27–31^. This in turn drive the dissociation of other host nuclear proteins death domain-associated protein (hDaxx) and ATP-dependent helicase (ATRX) from the viral genome, preventing them from repressing viral gene expression^32^. More recently, the E3 ligase Really Interesting New Gene (RING) finger domain of ICP0 has been shown to be responsible for proteasome-dependent degradation of several cellular proteins such as Nuclear domain 10 (ND10)^33^. After entering the cell, HSV-1 is confronted with early host defence transcriptional repression machinery including the corepressor of RE1 silencing transcription factor (CoREST) complex^34^. Interestingly it was found that in the presence of ICP0, 50% of the histone deacetylases that function in the CoREST complex dissociate, preventing transcriptional repression and enabling immune evasion^35–37^. Multiple studies have also shown that ICP0 inhibits IFN-I production resulting in diminished innate immune responses by inhibiting IRF3 regulated transcription^38,39^ and by inhibiting tumor necrosis factor alpha-induced NF-κB^40^, STING^41^ and IFI16^42^ activation.

The results from our investigations into understanding of the pathogenesis of HSK demonstrate that ICP0 inhibits type I IFN activity downstream of PRRs and cytosolic nucleic acid receptors which are known to be involved in host antiviral defences. Additionally, we have identified interferon regulatory factor (IRF)7, an important driver of IFN-I, as a target for ICP0 suggesting an additional immune evasion strategy for HSV-1.

## Results

### ICP0 acts downstream of RIG-I and TBK1 to negatively regulate activation of the IFN-β promoter

Production of IFN-I downstream of TLRs and cytosolic nucleic acid receptors is a key component of the innate immune systems antiviral response. HSV-1 has developed multiple mechanisms of evading host immune response by targeting different downstream components in order to promote viral replication. Our initial investigations focussed on identifying IFN-I pathways targeted by ICP0.

**Figure 1:**
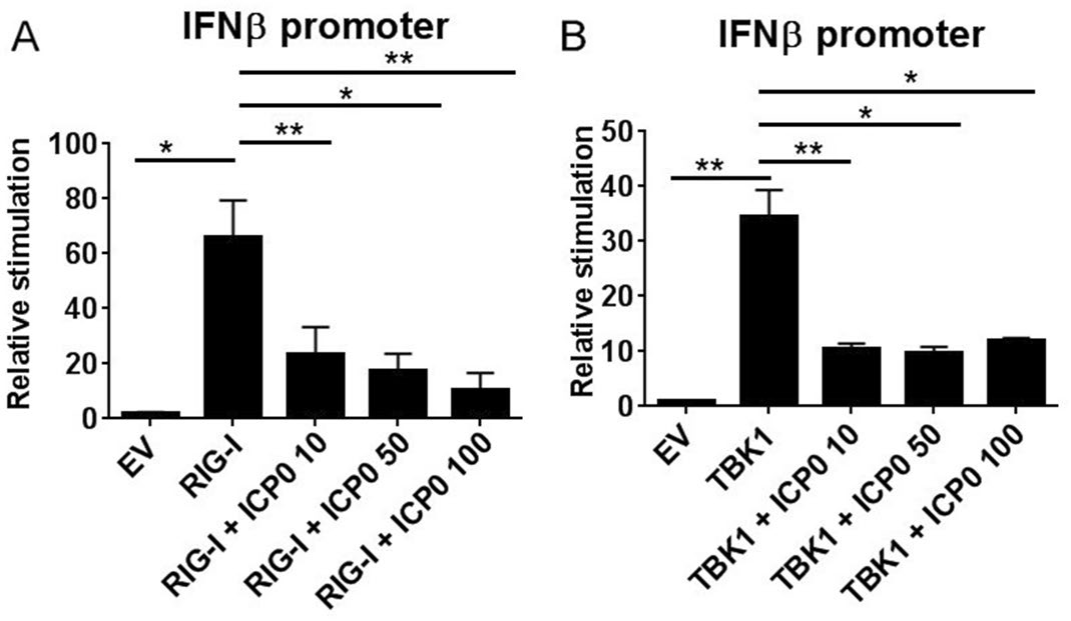
Full-length ICP0 negatively regulates IFN-β promoter activity in response to multiple drivers. HEK-293T cells were transiently transfected with 50 ng of the IFN-β p125 promoter, 5 ng of TK renilla, and increasing concentrations (10, 50 or 100 ng) of full-length ICP0 as indicated. In addition, cells were cotransfected with RIG-I (**A**) or TBK-1 (**B**) or empty vector (EV) control, as indicated, and assayed for reporter gene activity 18 h posttransfection. Results are expressed as the mean ± SD in each case are representative of three independent experiments expressed as fold stimulation over unstimulated empty vector (EV) control. *p < 0.05, **p < 0.01 as determined by Student *t* test.

To assess how ICP0 affected IFN-β expression we transfected HEK293T cells with increasing concentrations of ICP0 and assessed its effect on an IFN-β dependent reporter gene (p125-luciferase). In keeping with its role as a negative regulator of IFN-β induction, ICP0 significantly and dose dependently inhibited RIG-1 driven IFN-β promoter activity (Fig. 1A). Similar effects of ICP0 on MyD88 and TRIF driven IFN-β promoter activity were observed (Supplemental Figure 1A,B). Interestingly, ICP0 was also found to inhibit the ability of TBK1 driven IFN-β responses. As TBK1 is a critical kinase activated by RIG-I and TLR-3/4 in order to phosphorylate IRF3 and IRF7, these results strongly suggested that ICP0 may be acting at the level of TBK1 or potentially targeting transcription factors downstream (Fig. 1B).

**Figure 2:**
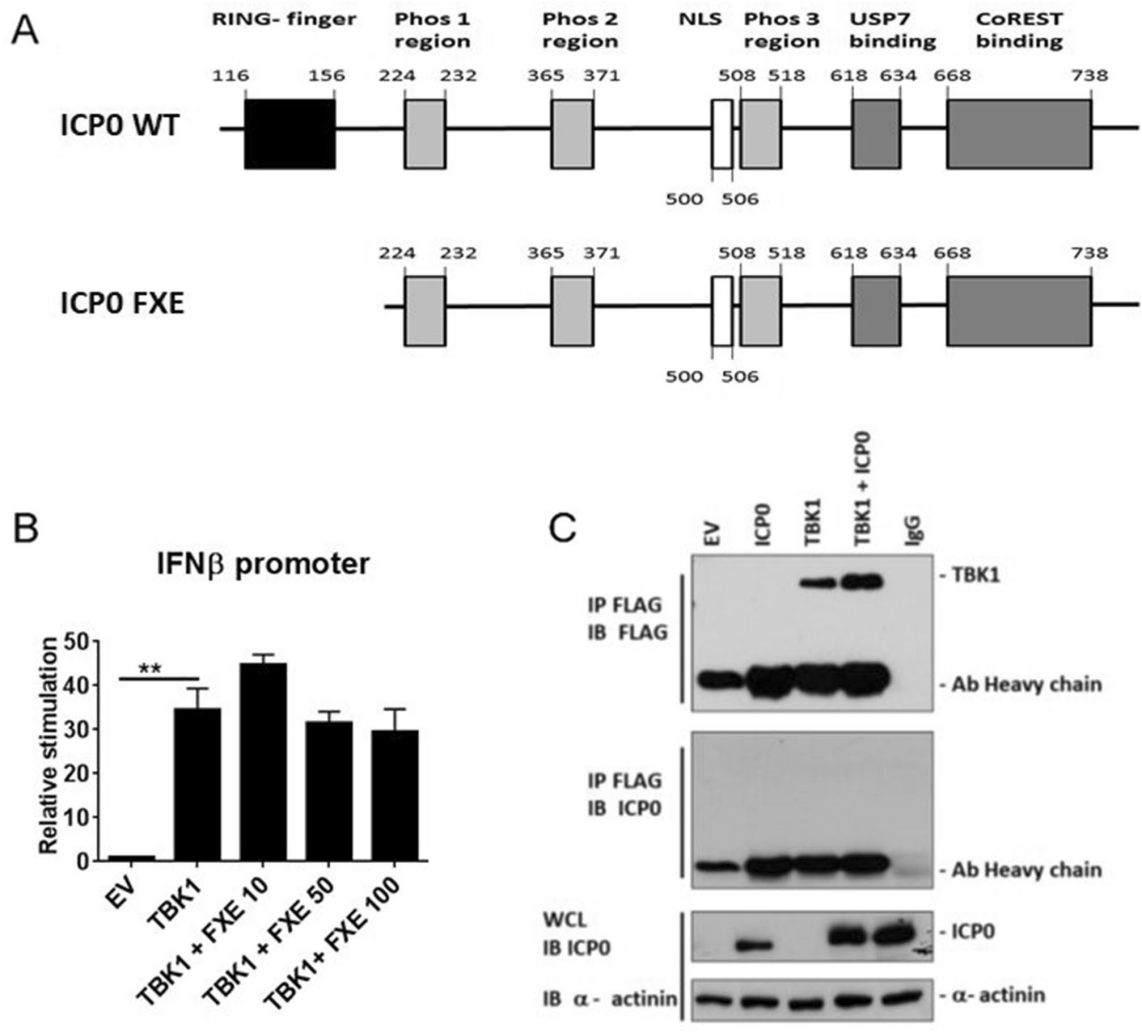
The RING finger domain of ICP0 is required for it to negatively regulate IFN-β promoter activity driven by TBK1. (**A**) A schematic diagram of wildtype ICP0 and its mutant (ICP0 FXE) lacking the RING-finger domain. Schematic created in Paint Shop Pro, version 5.01, https://www.paintshoppro.com. (**B**) HEK-293T cells were transiently transfected with a reporter construct containing the human IFN-β promoter. Cells were co-transfected with 50 ng of empty vector (EV) control or TBK1 and increasing amounts (10, 50 or 100 ng) of a plasmid encoding the RING finger domain deficient ICP0 (ICP0-FXE). The amount of DNA transfected into the cells was normalised using empty vector (EV). Cells were assayed for reporter gene activity 18 h posttransfection. Results are expressed as the mean ± SD in each case and are representative of three independent experiments expressed as fold stimulation over unstimulated empty vector (EV) control. **p < 0.01 as determined by Student *t* test. (**C**) 293T cells were transfected with constructs expressing the empty vector (EV), ICP0, FLAG-tagged TBK1 (lanes 1, 2 and 3) or ICP0 and TBK1 (lanes 4 and 5). Eighteen-hour posttransfection cell extracts were immunoprecipitated with an anti-FLAG antibody. Anti-IgG antibody was used in lane 5 for the control. Immunoprecipitated protein complexes were separated by SDS-PAGE and western blotted using anti-FLAG to detect TBK1 (panel 1) and anti-ICP0 antibody to detect ICP0 (panel 2). Presence of ICP0 was detected in whole cell lysates (WCL) by immunoblotting (panel 3). α-Actinin served as a loading control (panel 4). Results are representative of three independent experiments. The full gel image is included in the supplementary data.

**Figure 3:**
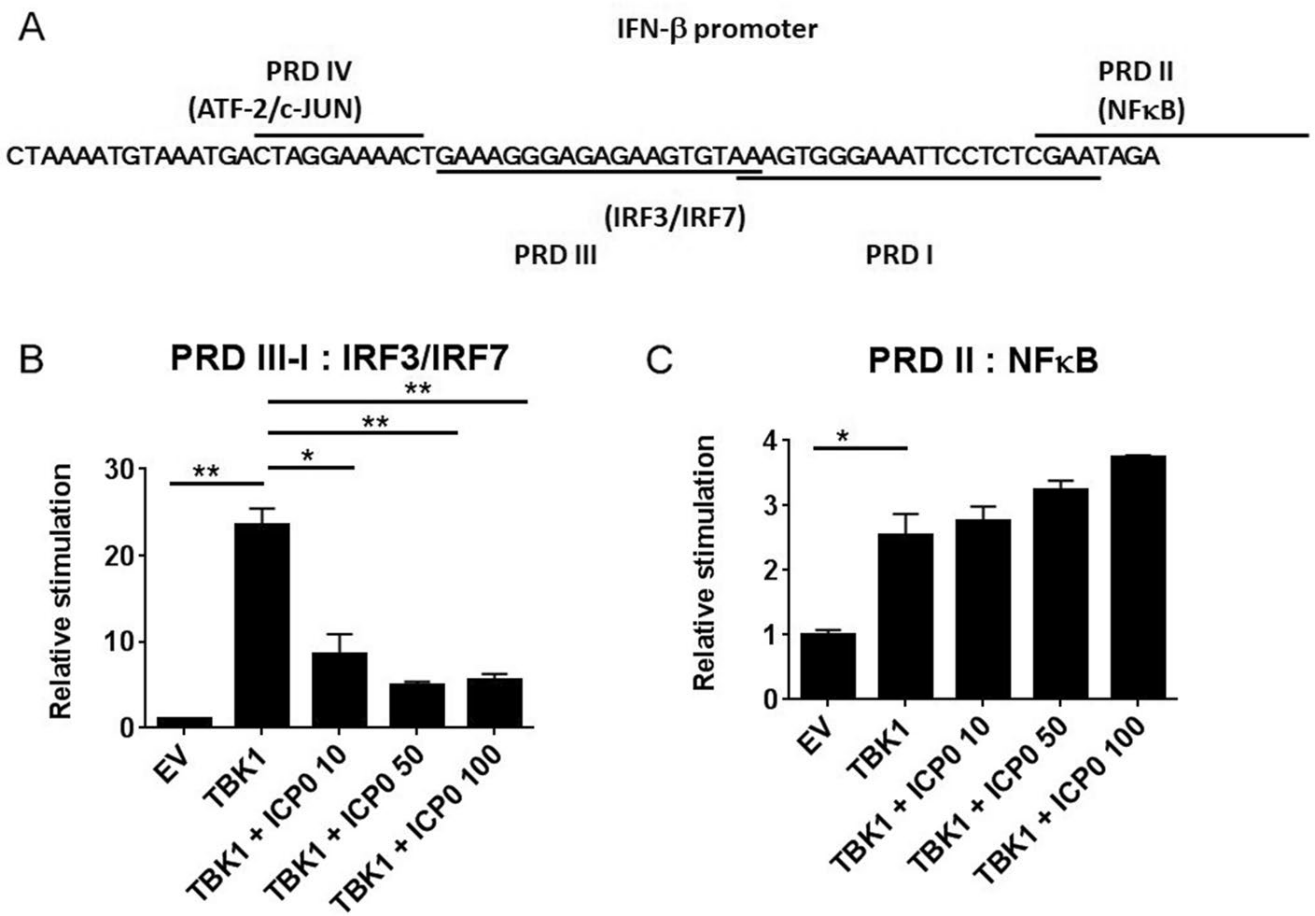
ICP0 negatively regulates TBK1 driven IFN-β promoter activity within the PRD III-I region. (**A**) A diagrammatic representation of the transcription factor binding sites in the IFN-β promoter. (**B**,**C**) HEK-293T cells were transiently transfected with reporter constructs containing PRD III-I-luc (**B**) or PRD II-luc (**D**) that encode the IRF3/IRF7 and NFκB regions of the IFN-β promoter respectively, together with 50 ng TBK-1 or empty vector (EV) control, as indicated, and increasing amounts (10, 50 or 100 ng) of ICP0–expressing construct. Cells were assayed for reporter gene activity 18 h posttransfection. Results are expressed as the mean ± SD in each case and are representative of three independent experiments expressed as fold stimulation over unstimulated empty vector (EV) control. *p < 0.05, **p < 0.01 as determined by Student *t* test.

Previous studies have shown that ICP0 has a RING finger domain that functions as an E3 ubiquitin ligase and is a crucial for many of ICP0’s properties (Fig. 2A). In order to determine whether the E3 ligase activity of ICP0 was responsible for these effects, we transfected HEK293T cells with increasing amounts of RING finger deficient ICP0 (ICP0-FXE) and TBK1 to assess its effect on the activation of the pathway. These studies demonstrated that RING finger-deficient ICP0 loses its suppressive action on IFN-β promoter activity when driven TBK1 (Fig. 2B), suggesting a role for ubiquitination dependent regulation of this pathway. In contrast, ICP0-FXE was still able to inhibit TRIF-driven IFN-β promoter activity, suggesting that the effects of ICP0 in regulating TRIF-driven responses in the cell are independent of any potentially E3 ligase activity ICP0 possesses (Supplemental Figure 1C). We next assessed a possible interaction between ICP0 and TBK1. FLAG-tagged TBK1 was immunoprecipitated from cells, however no interaction between ICP0 and TBK1 was detected (Fig. 2C). Given the lack of interaction between TBK1 and ICP0 our results suggested that ICP0 may be targeting IRF3/7 or possibly NFκB downstream of TBK1, which are required for IFN-β expression. In order to determine which of these transcription factor families may be targeted we utilized reporter gene constructs containing the IRF3/7 (PRDIII-I) or NFκB (PRDII) recognition sites in the IFN-β promoter (Fig. 3A). ICP0 significantly inhibited TBK-1-driven PRDIII-I-luciferase activity but not PRDII, indicating that the effects of ICP0 were targeting IRF3/IRF7 activation but not NFκB activity. Interestingly a slight potentiation of the TBK-1 driven PRDII response was observed (Fig. 3B,C).

### ICP0 regulates the stability of IRF7

**Figure 4:**
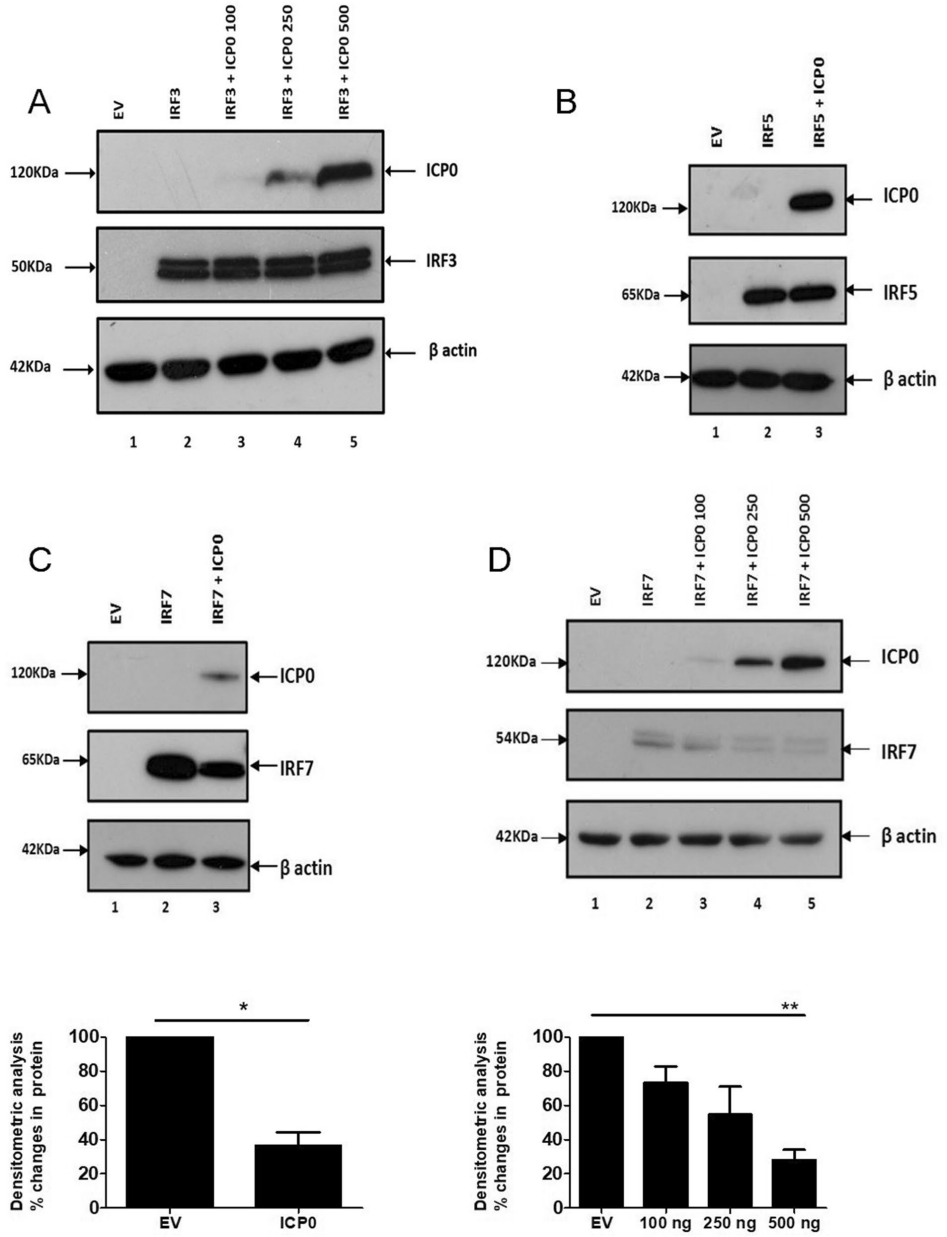
ICP0 induces destabilization of IRF7. (**A**) 293T cells were transfected with 100ug FLAG-tagged IRF3 and increasing amounts of ICP0 as indicated (100 ng, 250 ng and 500 ng in lanes 3, 4 and 5 respectively) or an EV control. Cell lysates were western blotted using anti-FLAG antibody to detect any change in the expression of IRF3 in the presence of ICP0. Presence of ICP0 was detected by immunoblotting (upper panel). The β-actin served as a loading control (lower panel). (**B**,**C**) 293T cells were transfected with constructs expressing the empty vector (EV), ICP0 or with key regulators of type 1 interferon pathway (**B**) Myc-tagged IRF5 or (**C**) Flag-tagged IRF7. Eighteen-hour posttransfection cell extracts were western blotted using anti-Myc or anti-FLAG antibodies to detect any change in the expression of target proteins. Presence of ICP0 was detected by immunoblotting (upper panels). β-Actin served as a loading control (lower panels). (**D**) 293T cells were transfected with 100 μg FLAG-tagged IRF7 and increasing amounts of ICP0 as indicated (100 ng, 250 ng and 500 ng in lanes 3, 4 and 5 respectively) or an EV control. Cell lysates were western blotted using anti-FLAG antibody to detect any change in the expression of IRF7 in the presence of ICP0. Presence of ICP0 was detected by immunoblotting (upper panel). The β-actin served as a loading control (lower panel). Results are representative of three independent experiments. Densitometric analysis was performed and graphs represent changes in total IRF7 protein levels relative to β-actin (**C**,**D**). Results are expressed as the mean ± SD in each case and are representative of three independent experiments expressed as IRF7 fold expression over β-actin. *p < 0.05, **p < 0.01 as determined by Student *t* test.

As there is some controversy in the literature regarding the role of ICP0 in regulating IRF3 activity we next performed stability experiments evaluating IRF3 degradation in the presence of increasing concentrations of ICP0^38,43^. We determined there was no evidence that IRF3 was being targeted by ICP0 for degradation (Fig. 4A). Similarly, no effects on the stability of TRAF3 (an adaptor protein downstream of RIG-I) was observed (Supplemental Figure 2A). In keeping with the role previously ascribed to ICP0 by Van Lint et al. we observed reduced expression of MyD88 in the presence of ICP0 (Supplemental Figure 2B)^44^. Next, we co-transfected HEK293T cells with ICP0 and IRF5 or IRF7. While the stability of IRF5 remained unchanged in the presence of ICP0 (Fig. 4B), there was a significant decrease in IRF7 protein levels in the presence of ICP0 (Fig. 4C). Furthermore our results show that ICP0 regulates the stability of IRF7 in a dose dependent manner (Fig. 4D). It is important to note that in each case potential targets were under control of a constitutive promoter, indicating that any effects of ICP0 on transfected protein levels represent the effect of ICP0 on protein stability rather than gene expression. Higgs et al. have demonstrated that TRIM21 targets IRF7 (in addition to IRF3 and IRF5) for degradation after the activation of viral recognition receptors^45^, which is of interest given that ICP0 is known to interact with other members of the TRIM family such as promyelocytic leukaemia (PML; also known as TRIM19) to disrupt host antiviral responses. To test whether ICP0 was utilizing TRIM21 to reduce IRF7 expression we tested their ability to interact in coimmunoprecipitation experiments. No interaction between ICP0 and TRIM21 was detected (data not shown), suggesting ICP0 is utilizing an unknown mechanism to regulate IRF7 stability.

## Discussion

There has been significant interest in determining the mechanisms used by HSV-1 to regulate IFN-I production given the central role it plays in limiting HSV-1 replication in the cornea as well as the systemic spread of infection^12^. This is the first study to demonstrate that ICP0 inhibits the production of IFN-I driven by IRF7. Furthermore, our co-transfection experiments have shown that expression of IRF7 is decreased by ICP0 in dose dependent fashion. These findings are another step towards a better understanding of the pathogenesis of HSK and may potentially identify an additional immune evasion strategy that is utilised by HSV-1.

Numerous HSV-1 viral proteins have been identified that contribute to evasion of the host immune response through an array of mechanisms including inhibiting NF-κB activation (UL24, UL42, UL36)^46–48^, modulating IRF3 (US3, VP16)^39,49^ or STING (VP22)^50^ function and consequently IFN-β production. The role of ICP0 is of particular interest as it has been dubbed as a “promiscuous transactivator” promoting expression of HSV-1 genes and pathogenesis, in addition to its role in aiding the virus to overcome innate immune responses^15–22^. Furthermore previous studies have shown that ICP0 diminishes the innate immune response to HSV-1 by inhibiting IRF3-, NF-κB-, STING- and IFI16-regulated pathways^38–42^. Of note Van Lint et al. have suggested that ICP0 exerts inhibitory effects on TLR2 signaling through the degradation of MyD88 and Mal and other adaptor or signaling molecules^51^. Our initial results are in line with their findings whereby we observed decreased expression of MyD88 by western blot when HEK293T cells were transfected with plasmids encoding MyD88 along with increasing concentrations of ICP0. In this study we uncovered a novel target for ICP0, the transcription factor IRF7 in regulating PRR-driven IFN-β expression.

Our initial investigations demonstrated that ICP0 inhibits RIG-I driven IFN-β promoter activity, potentially indicating that the effects of ICP0 are at a common point on both the TLR and RLR-driven pathways regulating IFN-β production. Studies have shown collaboration of TLRs and cytoplasmic RLRs for triggering antiviral innate immune responses^52–54^. Both pathways share crucial signalling factors, such as NF-kB, TBK1 and IRF3 that have already been implicated in the complex interplay between HSV-1 and hosts antiviral responses^41^. As TBK1 is a key downstream intermediate of these pathways we next investigated the potential that ICP0 could modulate TBK1 activity^55^. We observed that ICP0 could disrupt TBK1-mediated IFN responses particularly within the IRF3/IRF7 binding sites of the IFN-β promoter and consistent with previous studies we have shown that the E3 ligase activity of ICP0’s RING-finger domain is important for its suppressive action^56^. Together these results suggested that ICP0 may be functioning in an analogous manner to HSV-1 viral protein US11, which inhibits RIG-1 and STING- mediated activation via targeting TBK-1^57,58^. However we did not observe a direct interaction between ICP0 and TBK1 suggesting that ICP0 could potentially interfere with the activity of TBK1 by means of translocation rather than direct interaction, similarly to the way ICP0 inhibits IRF3 protein activity^59^.

Given then effects we observed on the different portions of the IFN-β we next investigated the interferon regulatory factor (IRF) family of transcription factors for potential interaction partners with ICP0. It has long been suggested that IRF7, the “master regulator” of IFN-I-dependent immune responses^60,61^ found in abundance on corneas of patients with a history of HSK, could be targeted by HSV-1 to overcome the innate antiviral response. The exact molecular mechanisms however were not clear^59^. An interesting study by Murphy et al.^62^ found the trigeminal ganglia of double deleted IRF3/7^−/−^ infected mice had significantly higher viral loads than wild-type or single knockout mice and suggests a synergistic control of HSV-1 pathogenesis by IRF3 and IRF7. Previous studies have examined the role of ICP0 in regulating IFN-Is via its effect on IRF3 and they report conflicting results. Some studies suggest that there is no direct interaction between ICP0 and IRF3^38^ and that loss of IRF3 does not affect the replication of HSV-1 in cultured cells^43^, while others suggest that ICP0 may instead be responsible for sequestering IRF3 or machinery required for IRF3 activation and IFN induction^42,63,64^. Consistent with Paldino et al.^38^ we did not observe a direct interaction between IRF3 and ICP0. In co-transfection experiments, we observed that expression of ICP0 culminated in reduced IRF7 expression. This is in keeping with the importance of IRF7 as a key target for immune evasion strategies as with other herpes viruses including Kaposi's sarcoma-associated herpesvirus (KSHV) and Epstein–Barr virus (EBV)^65,66^.

Although the treatment strategies vary depending on the type of keratitis, none of the current treatments is able to completely eliminate the virus, and thus the risk of recurrence always exists. Therefore, the optimal treatment is one that achieves the longest remission with minimal local or systemic side effects. The antiviral agents acyclovir, ganciclovir and trifluridine are effective against the active virus, but do not eliminate the latent infection. Long term topical and systemic use is associated with toxicity and adverse effects^67,68^. Similarly, topical steroids, although important therapeutic agents in the management of HSK are associated with risks such as the development of cataract and glaucoma^69–71^ as well as facilitation of viral penetration into the cornea^72,73^. In advanced or recurrent cases, long-standing corneal inflammation can result in permanent scarring and loss of corneal transparency and is the major cause of decreased vision associated with HSK. Once this has occurred, the only viable treatment becomes corneal transplantation, a procedure that has a dismal long-term visual outcome for this clinical indication with regard to graft survival and an increased risk of recurrence of infection and therefore vision^74,75^.

Therefore more targeted and effective treatments of this potentially blinding condition are necessary. In this study we have shown that HSV-1 viral protein ICP0 reduces IRF7 protein expression thereby inhibiting IFN-I responses. IRF7 represents a critical common signalling molecule downstream of RLR, cytosolic nucleic acid sensor and TLR pathways. There have been several reports in the literature of successful treatment of recalcitrant herpetic infection in HIV patients with topical 5% Imiquimod, which is a known TLR7 ligand that activates the TLR7 pathway via IRF7^76–79^. However, none of these studies investigated the use of Imiquimod in ocular herpetic infection and even though the pathogenesis of the herpetic infections in different parts of the human body is largely similar, its effectiveness and safety profile for the use in herpetic keratitis remains unanswered. A limited number of studies reported the use of topical Imiquimod in a variety of other ocular and periocular conditions such as conjunctival actinic keratosis, periocular actinic keratosis and basal cell carcinoma^80^ however conjunctivitis and ocular stinging were encountered as adverse effects that resolved on termination of Imiquimod therapy. As these effects were temporary further research is warranted for its potential role in the eye.

The importance of the adaptor proteins TRIF, RIG-I, TBK1 and MyD88 in the pathogenesis of viral infections has been well established^9,41,44,57,81–87^. In our study we demonstrated the significant inhibitory effect of the multifunctional HSV-1 regulatory protein ICP0 on type I IFN responses driven by these proteins which represent key downstream effector molecules of several TLRs (3, 4 and 7) in addition to RLR and cytosolic nucleic sensing pathways, consistent with suggestions that HSV-1 targets common components of these pathways to overcome cross regulation and immune detection^52–54^. In the search for novel targets for ICP0, we have identified IRF7. These findings may help pave the way in better understanding the pathogenesis of this blinding condition and develop more efficient treatment options to reduce the morbidity and improve the quality of lives of the affected patients.

## Materials and methods

### Culture of cell lines

As previously described HEK293T cells were cultured in Dulbecco’s Modified Essential Medium (DMEM) high glucose containing stable 2 mM l-glutamine, 10% (v/v) heat inactivated and filtered sterilised foetal calf serum (FCS) and 100 units/ml Penicillin/100 µg/ml Streptomycin. Cells were maintained at 37 °C in a humidified atmosphere of 5% CO_2_. For use in transfection assays, HEK293T cells were typically seeded at 2.5–3 × 10^5^ cells/ml 24 h prior to transfection^88^.

### Luciferase reporter gene assays

As previously described HEK293T cells (purchased from ATCC, Middlesex, U.K.) were seeded at a density of 2 × 10^5^/ml (final volume of 200 μl) and were transiently transfected with combinations of plasmids (indicated in the figure legends), using Metafectene (Biontex, Martinsried, Germany) according to the manufacturer’s recommendations^88^. We examined the expression of luciferase 18 h after the initial cell transfection. The supernatant was removed and HEK 293T cells were lysed. The appropriate substrate was added to the lysed cells (luciferin or coelenterazine) and the luminescence was determined. The amount of light produced therefore provided a quantitative measure of the effect of ICP0 on expression of the various target genes. Luciferase activity was normalised to *Renilla* luciferase plasmid activity to normalise for transfection efficiency. Results are expressed as mean relative stimulation from three separate experiments ± SD.

### Plasmids and reagents

Flag-tagged pCMV-IRF3, pEF-*Bos*-TRIF-Flag, flag-tagged TBK1, and the IFN-β promoter constructs were from Dr. Kate Fitzgerald (University of Massachusetts Medical School, Worcester, MA). WT ICP0 and ICP0-FXE were kind gifts from Professor Roger Everett (University of Glasgow, Centre of Virus Research). Xpress tagged TRIM21 was a gift from Dr. David Rhodes (Cambridge Institute for Medical Research, Wellcome Trust/MRC Building, Addenbrooke’s Hospital Hills Road, Cambridge CB2 2XY, UK).

### Antibodies

Primary antibodies used were anti-TBK1 (Alexis Biochemicals, Lausen, Switzerland), anti-TRIF (Abnova, Walnut, CA), anti–TNFR-associated factor 3 (TRAF3) (Santa Cruz Biotechnoly), and anti-β-actin (Abcam, Cambridge, U.K.), anti-IRF3 (Santa Cruz Biotechnologies), anti-Flag (Sigma-Aldrich), anti-Xpress (Invitrogen Life), Anti-α-Actinin (H-300) (Santa Cruz Biotechnologies), anti-ICP0 (Santa Cruz Biotechnologies), anti-IRF5 (Cell Signalling), anti-IRF7 (Abcam).

### Western blot and immunoprecipitation analysis

Immunoblots were performed as described previously^29^. Cells were lysed on ice in 1 × radioimmunoprecipitation lysis buffer (1 × PBS, 1% Nonidet P-40, 0.5% Na-deoxycholate, 0.1% SDS, 1 mM KF, 1 mM Na_3_VO_4_, 10 μg/ml leupeptin, and 1 mM PMSF) followed by immunoprecipitation with anti-TBK1 or anti-TRAF3 precoupled to protein-G Sepharose beads.

For Western blots the molecular weight ladder employed had bands at 170, 130, 100, 70, 55, 40, 35 and 10 kDa. We routinely cut the Western blot membrane just below the 55 kDa band following blotting for IRF7 and TRAF3 and have used this portion to blot for the loading control. As such the image presented in figures represents the full length gel for loading controls. All Western blot figures were created in Paint Shop Pro, version 5.01, https://www.paintshoppro.com.

### Statistical analysis

Data were analysed using Prism 6 software, version 6.07, https://www.graphpad.com/ (GraphPad Software, La Jolla, CA, USA). The Students paired t test was performed to examine changes in luciferase promoter activity. Data was deemed significantly different at *P* values less than 0.05.

## Supplementary information

Supplementary Figures.
