# Supplementary material for: Herpes simplex virus 1 targets IRF7 via ICP0 to limit type I IFN induction
Source: Sci Rep. 2020 Dec 17;10:22216. doi: 10.1038/s41598-020-77725-4 (PMC7747705; doi:10.1038/s41598-020-77725-4)
Supplement: Supplementary file 1 — Supplementary Figures. [file 41598_2020_77725_MOESM1_ESM.docx]

**Herpes simplex virus 1 targets IRF7 via ICP0 to limit type I IFN** **induction**

David Shahnazaryan (1,2), Rana Khalil (3), Claire Wynne (4), Caroline A. Jefferies^#^ (5), Joan Ní Gabhann-Dromgoole^#^ (1, 3) and Conor C. Murphy^#^ (1,2)

^#^Authors contributed equally

(1) Department of Ophthalmology, Royal College of Surgeons in Ireland, Dublin 2, Ireland.

(2) Department of Ophthalmology, Royal Victoria Eye and Ear Hospital, Dublin 2, Ireland. (3) School of Pharmacy and Biomolecular Sciences (PBS) and RSCI Research Institute, Royal College of Surgeons in Ireland, Dublin 2, Ireland.

(4) Technological University (TU) Dublin, School of Biological and Health Sciences, Kevin Street, Dublin 8, Ireland

(5) Division of Rheumatology, Department of Medicine and Department of Biomedical Sciences, Cedars-Sinai Medical Centre, 8700 Beverly Blvd, Los Angeles, California 90048, USA.

Please address correspondence to **Dr Joan Ní Gabhann-Dromgoole**, School of Pharmacy and Biomolecular Sciences (PBS) and RSCI Research Institute, Royal College of Surgeons in Ireland, Dublin 2, Ireland. Tel: 33 01 4025216. Email : [joannigabhann@rcsi.ie](mailto:joannigabhann@rcsi.ie)

**
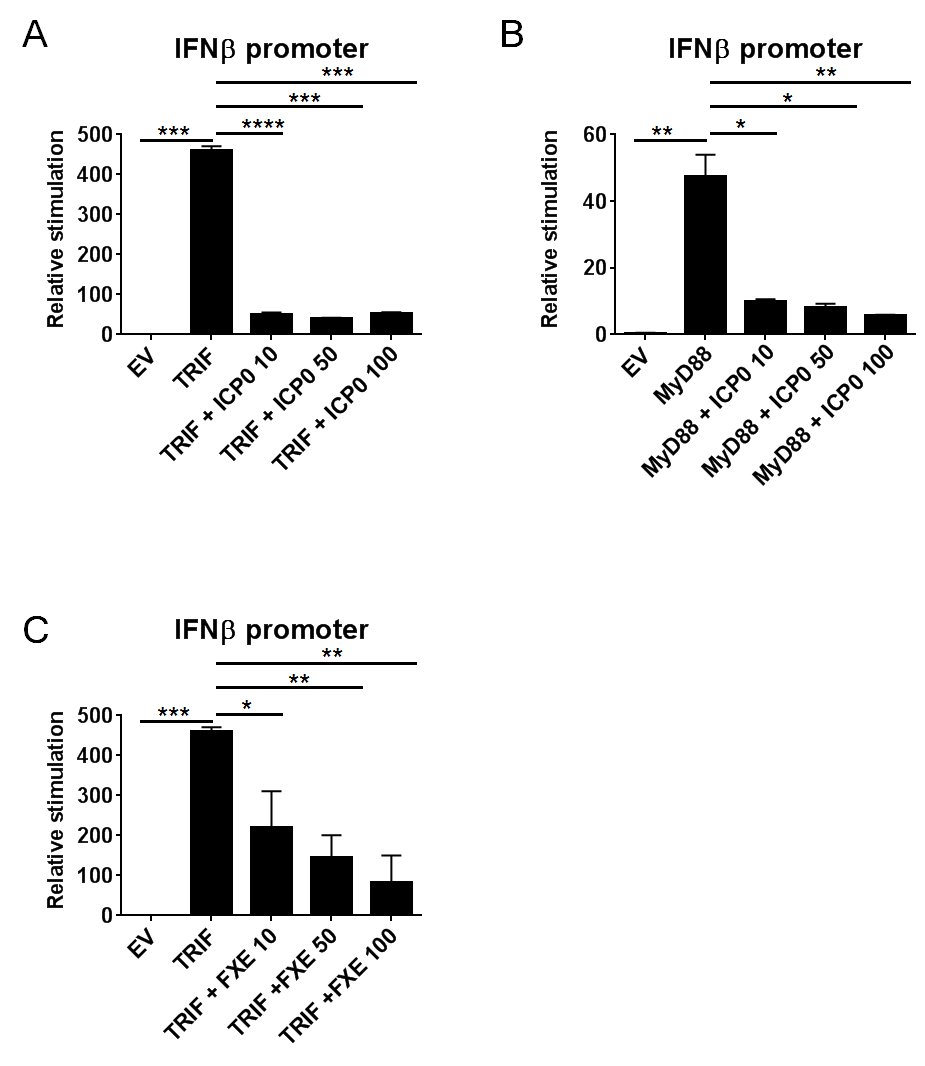
**

**Supplemental Figure 1: Full-length ICP0 negatively regulates IFN-β promoter activity.**

HEK-293T cells were transiently transfected with 50ng of the IFN-β p125 promoter, 5ng of TK renilla, and increasing concentrations (10, 50, or 100 ng) of full-length ICP0 as indicated. In addition, cells were cotransfected with TRIF (*A*), MyD88 (*B*) or empty vector (EV) control, as indicated, and assayed for reporter gene activity 18 h posttransfection. Results in each case are representative of three independent experiments. *(C)* HEK-293T cells were transiently transfected with a reporter construct containing the human IFN-β promoter. Cells were co-transfected with 50 ng of empty vector (EV) control or TRIF and increasing amounts (10, 50 or 100 ng) of a plasmid encoding the RING finger domain deficient ICP0 (ICP0-FXE). In all cases, results are expressed as the mean ± SD in each case and are representative of three independent experiments expressed as fold stimulation over unstimulated empty vector (EV) control.. **p* < 0.05, ** *p* < 0.01, *** *p* < 0.001 and *****p* < 0.0001 as determined by Student *t* test.


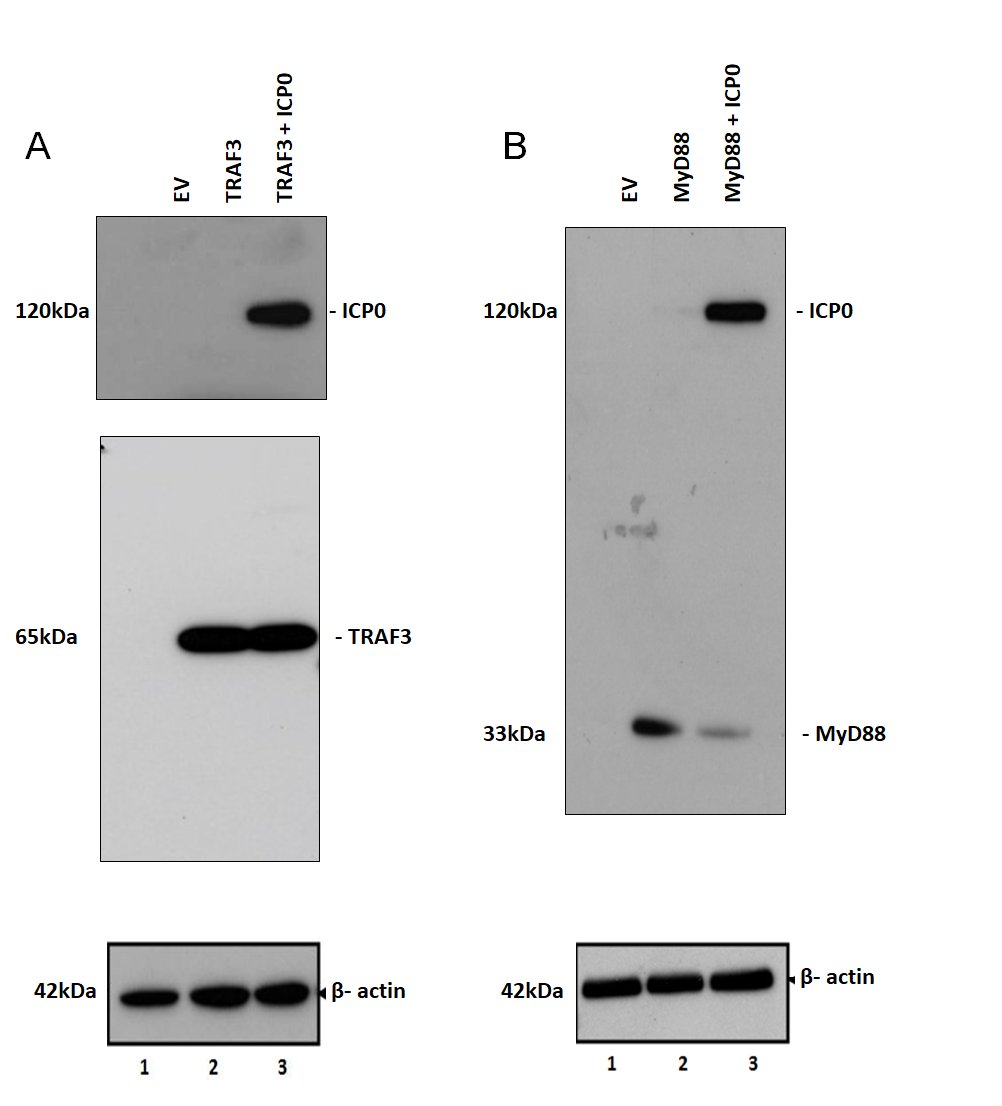


**Supplemental Figure 2. Reduced MyD88 expression in the presence of ICPO.**

293T cells were transfected with constructs expressing the empty vector (EV), ICP0 or with key regulators of type 1 interferon pathway FLAG-tagged TRAF3 or MyD88 as indicated. Eighteen-hour posttransfection cell extracts were western blotted using anti-FLAG (A) or anti-MyD88 (B) antibodies to detect any change in the expression of target proteins. Presence of ICP0 was detected by immunoblotting (upper panels). β-actin served as a loading control (lower panels). .


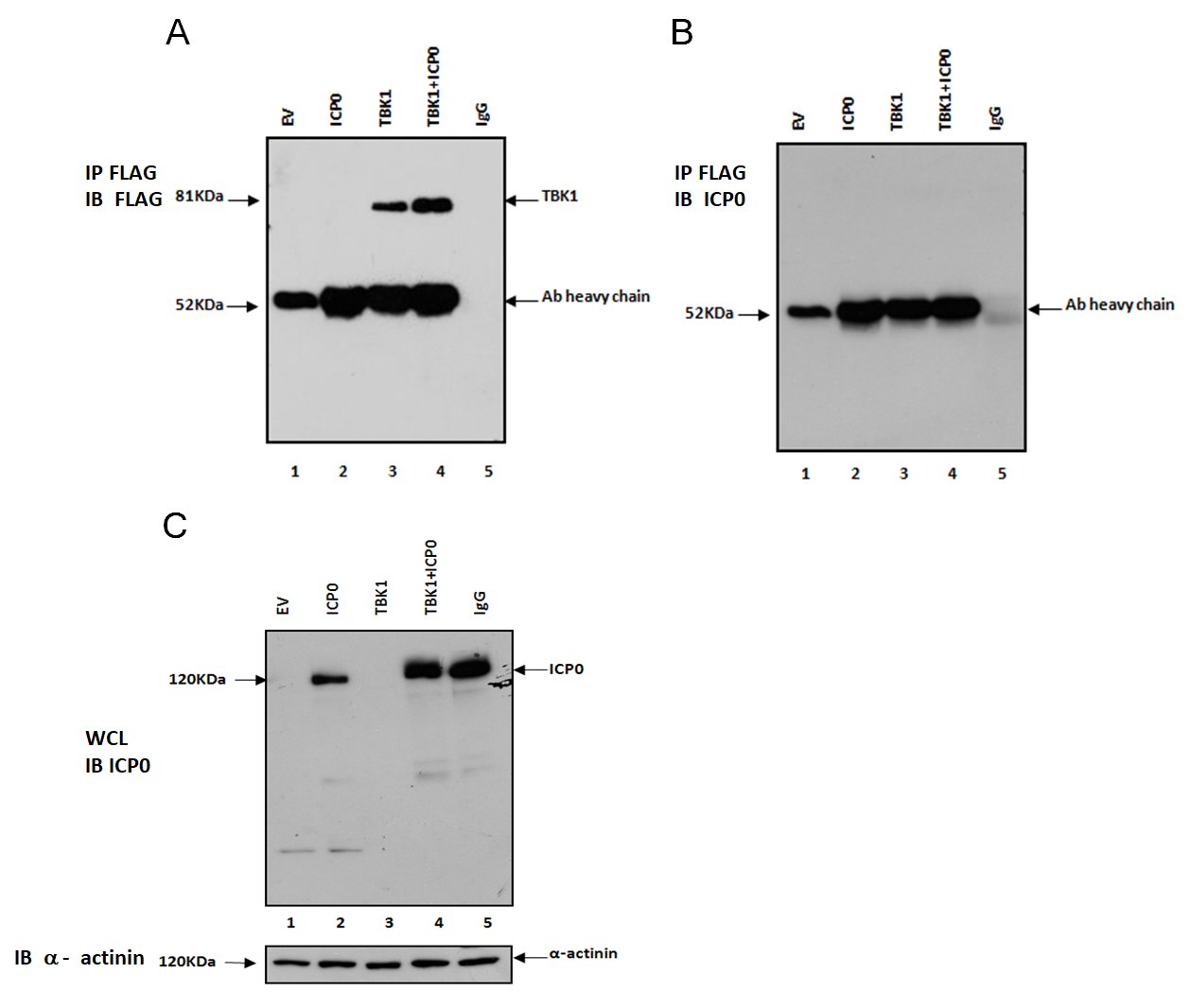


**Supplemental Figure 3. ICP0 dose not interact with TBK1.** 293T cells were transfected with constructs expressing the empty vector (EV), ICP0, FLAG-tagged TBK1 (lanes 1, 2 and 3) or ICP0 and TBK1 (lanes 4 and 5). Eighteen-hour posttransfection cell extracts were immunoprecipitated with an anti-FLAG antibody. Anti-IgG antibody was used in lane 5 for the control. Immunoprecipitated protein complexes were separated by SDS-PAGE and western blotted using (A) anti-FLAG to detect TBK1 and (B) anti-ICP0 antibody to detect ICP0. (C) Presence of ICP0 was detected by immunoblotting (upper panel). α-actinin served as a loading control (Lower panel). Results are representative of three independent experiments.

**
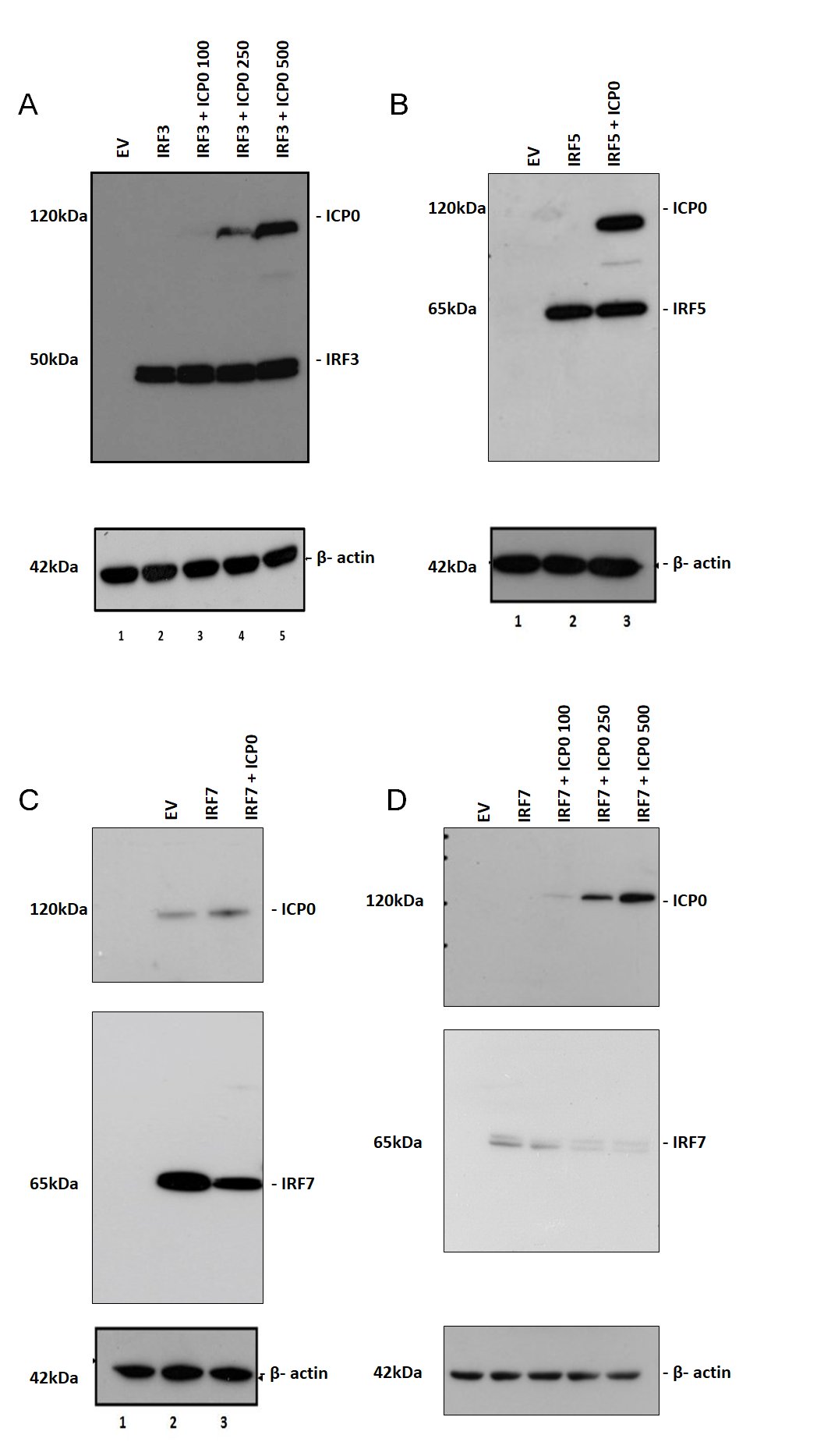
**

**Supplemental Figure 4. ICP0 induces destabilization of IRF7.** (*A*) 293T cells were transfected with 100ug FLAG-tagged IRF3 and increasing amounts of ICP0 as indicated (100ug, 250ug and 500ug in lanes 3, 4 and 5 respectively) or an EV control. Cell lysates were western blotted using anti-FLAG antibody to detect any change in the expression of IRF3 in the presence of ICP0. Presence of ICP0 was detected by immunoblotting (upper panel). The β-actin served as a loading control (lower panel). (*B-C*) 293T cells were transfected with constructs expressing the empty vector (EV), ICP0 or with key regulators of type 1 interferon pathway (B) Myc-tagged IRF5 or (*C*) Flag-tagged IRF7. Eighteen-hour posttransfection cell extracts were western blotted using anti-Myc or anti-FLAG antibodies to detect any change in the expression of target proteins. Presence of ICP0 was detected by immunoblotting (upper panels). β-actin served as a loading control (lower panels). (*D*) 293T cells were transfected with 100ug FLAG-tagged IRF7 and increasing amounts of ICP0 as indicated (100ug, 250ug and 500ug in lanes 3, 4 and 5 respectively) or an EV control. Cell lysates were western blotted using anti-FLAG antibody to detect any change in the expression of IRF7 in the presence of ICP0. Presence of ICP0 was detected by immunoblotting (upper panel). The β-actin served as a loading control (lower panel). Results are representative of three independent experiments.
